# Postinfluenza Organizing Pneumonia in an Older Adult Successfully Treated With Corticosteroids: A Case Report

**DOI:** 10.7759/cureus.107840

**Published:** 2026-04-27

**Authors:** Natsumi Yamamoto, Ryuichi Ohta, Akira Yamasaki, Chiaki Sano

**Affiliations:** 1 Community Care, Unnan City Hospital, Unnan, JPN; 2 Multidisciplinary Internal Medicine, School of Medicine, Faculty of Medicine, Tottori University, Yonago, JPN; 3 Community Medicine Management, Shimane University Faculty of Medicine, Izumo, JPN

**Keywords:** adrenal cortex hormones, aged, computed tomography, general medicine, influenza a virus infection, interstitial, organizing, pneumonia, rural

## Abstract

Influenza infection is usually self-limiting; however, it may occasionally trigger secondary inflammatory lung diseases such as organizing pneumonia, particularly in older adults. An 89-year-old man presented with progressive anorexia and dyspnea after a recent influenza A infection. Although he was initially managed conservatively, his respiratory symptoms worsened after a transient improvement. On admission, he had tachypnea, hypoxemia, bilateral crackles, hypoalbuminemia, elevated inflammatory markers, and mild liver dysfunction. Chest radiography and computed tomography (CT) demonstrated bilateral subpleural infiltrative opacities distributed along the bronchovascular bundles, with small pleural effusions. Based on the clinical course and imaging findings, postinfluenza organizing pneumonia was suspected. The patient was treated with systemic corticosteroids and antibiotics, followed by gradual steroid tapering. His respiratory symptoms and general condition improved steadily, and he was discharged home on hospital day 14. Follow-up CT performed one month after discharge showed marked improvement in the bilateral pulmonary infiltrates, and his functional status returned to baseline. This case highlights that persistent or worsening respiratory symptoms after influenza infection in older adults may indicate post-infectious organizing pneumonia rather than simple secondary bacterial pneumonia and that early recognition and corticosteroid treatment may contribute to favorable clinical recovery and prevention of functional decline.

## Introduction

Influenza is a common systemic viral infection that can cause acute organ damage [1]. While most influenza infections are self-limiting and managed with supportive care, severe cases may require antiviral treatment to prevent complications [2]. However, influenza can also trigger persistent systemic inflammation, leading to secondary inflammatory diseases such as interstitial pneumonia [3]. These complications may present after the acute phase of infection and can progress rapidly, particularly in vulnerable populations such as older adults [1,4]. Recognizing and managing postinfluenza inflammatory complications remains a clinical challenge due to their nonspecific presentation.

In this report, we present the case of an elderly patient who developed persistent fever following an influenza infection and was subsequently diagnosed with interstitial pneumonia. Postinfluenza organizing pneumonia is rare, and its early recognition can be challenging because symptoms may initially be attributed to prolonged viral infection or secondary bacterial pneumonia. This case highlights the importance of considering organizing pneumonia in older adults with persistent respiratory symptoms and inflammatory findings after influenza infection.

## Case presentation

An 89-year-old man presented with progressive anorexia and dyspnea following a recent influenza infection. He had previously been independent in activities of daily living and lived with his wife and his son’s family. His past medical history included type 2 diabetes mellitus without pharmacological treatment and benign prostatic hyperplasia. He was a former smoker and consumed approximately one serving of shochu daily.

According to his family, the patient initially developed a headache and dizziness two weeks before admission. Ten days before admission, he visited another hospital and was diagnosed with influenza A. No antiviral medication was prescribed, and he was managed conservatively at home. Seven days before admission, he developed labored breathing and revisited the same hospital; however, due to a shortage of available beds, he was managed as an outpatient and prescribed symptomatic medications, including inhaled procaterol, ambroxol 45 mg, and acetaminophen 500 mg daily. His respiratory symptoms temporarily improved. However, after completing the medications two days before admission, he again experienced dyspnea and a sensation of mental clouding. On the day of admission, he revisited the hospital and was found to have pneumonia on imaging; therefore, he was referred to our institution for further management.

On admission, his vital signs were as follows: consciousness clear, blood pressure 127/92 mmHg, pulse rate 92/min, respiratory rate 24/min, body temperature 36.4°C, SpO_2_ 93% (room air). Physical examination revealed facial flushing and erythema on the anterior chest. Lung auscultation demonstrated late inspiratory crackles over the right anterior chest and bilateral lung bases. Cardiac examination revealed a regular rhythm without murmurs. The abdomen was soft and non-tender. Bilateral lower extremity edema was present.

Laboratory findings showed a white blood cell count of 7,370/µL, hemoglobin level of 15.4 g/dL, and platelet count of 243,000/µL. Serum albumin was markedly decreased at 2.0 g/dL. Liver enzymes were mildly elevated (aspartate aminotransferase (AST) 116 U/L, alanine aminotransferase (ALT) 89 U/L), and C-reactive protein was elevated at 7.4 mg/dL. Given the patient’s history of hepatitis C virus-related liver cirrhosis, these abnormalities were considered to reflect underlying chronic liver disease and acute inflammation due to pneumonia. Total bilirubin was within the normal range, and no clinical features suggested biliary obstruction or heart failure. Liver enzymes were monitored during admission. Renal function was preserved, with a serum creatinine level of 0.87 mg/dL. N-terminal pro-B-type natriuretic peptide (NT-proBNP) was 947 pg/mL (Table 1).

**Table 1:** Initial laboratory findings and urinalysis on admission This table summarizes the patient’s hematological, biochemical, inflammatory, and urinary findings at the time of hospital admission, together with the corresponding institutional reference ranges. CRP, C-reactive protein; KL-6, Krebs von den Lungen-6; Na, sodium; K, potassium; Cl, chloride

Parameter	Level	Reference
White blood cells	7.37	3.5-9.1×10^3^/μL
Neutrophils	72.9	44.0-72.0%
Lymphocytes	18.8	18.0-59.0%
Hemoglobin	15.4	11.3-15.2 g/dL
Hematocrit	47.6	33.4-44.9%
Mean corpuscular volume	99.2	79.0-100.0 f
Platelets	24.3	13.0-36.9×10^4^/μL
Total protein	6.2	6.5-8.3 g/dL
Albumin	2.0	3.8-5.3 g/dL
Total bilirubin	1.3	0.2-1.2 mg/dL
Aspartate aminotransferase	116	8-38 IU/L
Alanine aminotransferase	89	4-43 IU/L
Lactate dehydrogenase	395	121-245 U/L
Blood urea nitrogen	12.6	8-20 mg/dL
Creatinine	0.87	0.40-1.10 mg/dL
Serum Na	135	135-150 mEq/L
Serum K	4.0	3.5-5.3 mEq/L
Serum Cl	100	98-110 mEq/L
Ferritin	683	14.4-303.7 ng/mL
CRP	7.44	<0.30 mg/dL
KL-6	453	105-401 U/mL
Urine test	-	-
Leukocyte	Negative	Negative
Protein	Negative	Negative
Blood	Negative	Negative

**Figure 1:**
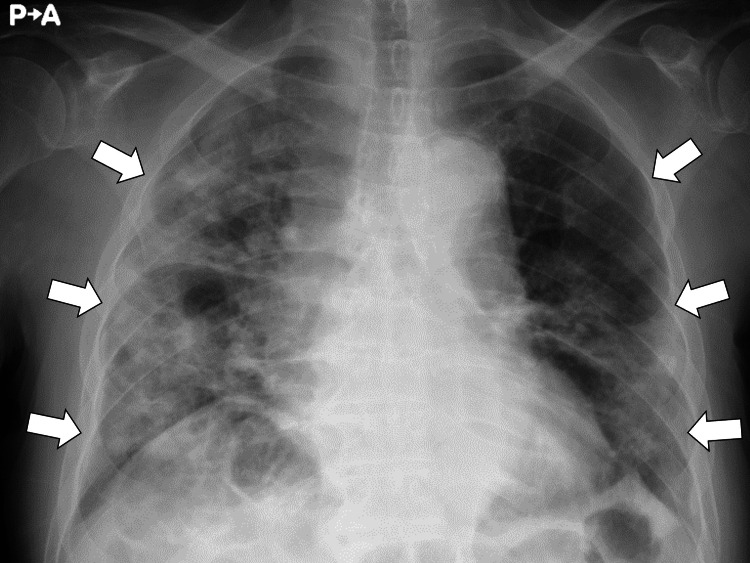
Chest radiography demonstrating bilateral subpleural infiltrative shadows (white arrows)

Chest radiography demonstrated bilateral subpleural infiltrative shadows (Figure 1).

**Figure 2:**
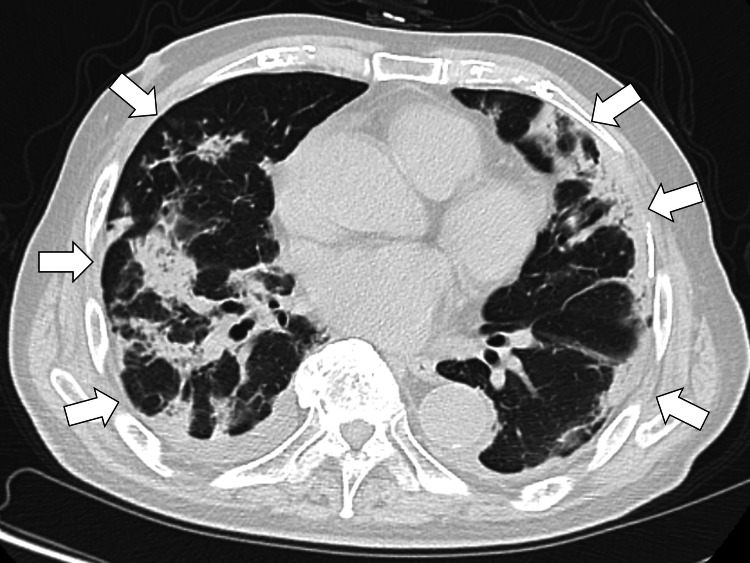
Chest CT showing multiple infiltrative opacities predominantly distributed in the subpleural regions and along the bronchovascular bundles, accompanied by bronchiectatic changes and small bilateral pleural effusions (white arrows) CT, computed tomography

Chest computed tomography (CT) showed multiple infiltrative opacities predominantly distributed in the subpleural regions and along the bronchovascular bundles, accompanied by bronchiectatic changes and small bilateral pleural effusions (Figure 2).

The key diagnostic clues in this case were the delayed worsening after apparent recovery from influenza A infection, persistent hypoxemia, elevated inflammatory markers, bilateral crackles, and bilateral subpleural infiltrates on CT. These findings were less typical of simple bacterial pneumonia, particularly because the clinical course was subacute and the radiological abnormalities were bilateral and peripheral. Therefore, postinfectious organizing pneumonia was considered. Based on these findings, viral pneumonia associated with recent influenza infection was suspected, although bacterial pneumonia and other interstitial lung diseases were considered as differential diagnoses.

Treatment was initiated with systemic corticosteroids and antibiotics. Intravenous hydrocortisone (200 mg/day) was started on January 18, along with ceftriaxone. The corticosteroid regimen was subsequently tapered as follows: methylprednisolone 40 mg for three days starting on day 2 of admission, followed by oral prednisolone 40 mg, gradually reduced to 20 mg, 10 mg, and 5 mg weekly.

**Figure 3:**
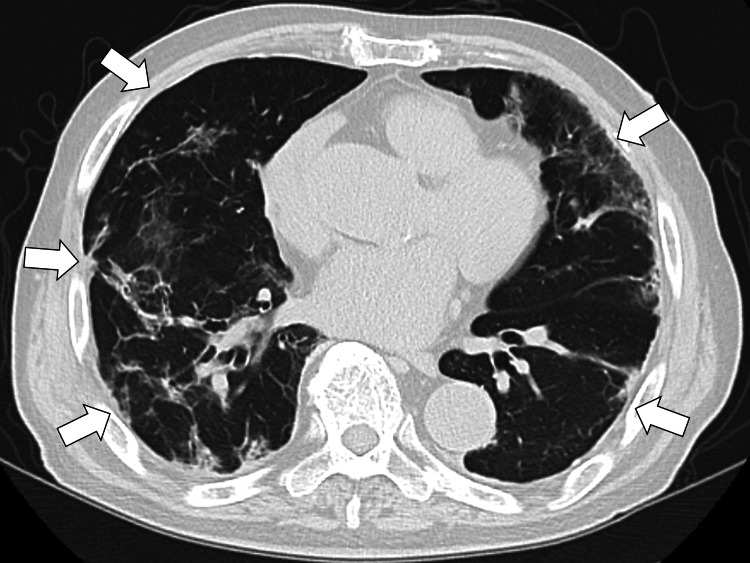
Chest CT one month after discharge showing improvement of the infiltration of both lungs (white arrows) CT, computed tomography

During hospitalization, glycemic control deteriorated, likely due to corticosteroid therapy, and pharmacological treatment for diabetes mellitus was initiated. Metformin was started on day 7 of admission, followed by empagliflozin on day 14. Hyperlipidemia was also treated with rosuvastatin 5 mg daily. Urine cytology for atypical cells was negative, and follow-up with urology was arranged. The patient’s respiratory condition gradually improved with treatment, and his clinical course remained stable; he was discharged home on day 14 after admission. One month after discharge, his condition returned to his usual condition, and the infiltration of both lungs on chest CT improved (Figure 3).

In six months, his prednisolone was tapered and stopped without any flare.

## Discussion

This case describes an 89-year-old man who developed persistent dyspnea and bilateral subpleural and peribronchovascular infiltrates after influenza A infection and who improved clinically and radiologically after corticosteroid therapy. The main learning points are that organizing pneumonia should be considered when respiratory symptoms worsen or fail to resolve after influenza, and that advanced age and metabolic vulnerability may contribute to progression to postinfectious inflammatory lung disease. In the present case, the subacute course after influenza, the bilateral nonlobar distribution on CT, and the favorable response to corticosteroids were all compatible with postinfluenza organizing pneumonia rather than uncomplicated bacterial pneumonia.

The first important point is the clinical behavior of organizing pneumonia after influenza. Postinfluenza organizing pneumonia appears to be uncommon, and the literature remains dominated by case reports, small series, and a limited systematic review rather than population-based studies, so its true incidence remains uncertain [5,6]. Published reports suggest that organizing pneumonia often emerges during the recovery phase of influenza or after apparent transient improvement, rather than at the very onset of viral illness [7]. Whether early antiviral treatment for influenza reduces the risk of subsequent organizing pneumonia remains unclear. In the present case, the patient had a recent influenza A infection but did not receive antiviral therapy. This temporal pattern is clinically important because such patients are easily misclassified as having persistent viral pneumonia or secondary bacterial pneumonia. Compared with bacterial superinfection, organizing pneumonia is more suggestive when symptoms evolve subacutely, imaging shows bilateral patchy infiltrates with a subpleural and/or peribronchovascular distribution, and there is a poor or incomplete response to antimicrobial therapy [8]. In the present case, the patient deteriorated after initial symptomatic treatment, and CT showed a distribution pattern well described in organizing pneumonia.

The second important point is identifying patients who may be at risk of developing organizing pneumonia after influenza. Influenza-specific risk factors for secondary organizing pneumonia have not been clearly established because the condition is rare, but prior literature supports a biologically plausible role for advanced age, comorbid diabetes, severe inflammatory lung injury, and hypoxemia [9]. In viral pneumonia-associated organizing pneumonia, older age and diabetes have been associated with postinfectious organizing phenotypes, although much of this evidence comes from broader viral pneumonia cohorts rather than influenza alone [10]. In the present case, the patient was very elderly, had untreated type 2 diabetes, and had ongoing respiratory compromise after influenza, all of which may have lowered the threshold for dysregulated repair and secondary organization. Although advanced age may contribute to high mortality, prompt diagnosis and treatment of interstitial pneumonia can mitigate morbidity and mortality, especially in rural contexts [11,12]. Frailty may also increase the risk of hospitalization and complications after influenza in older adults [12]. Therefore, frailty assessment may help clinicians evaluate risk more objectively. However, because these factors are not yet specific predictors of influenza-associated organizing pneumonia, they should be interpreted cautiously as risk markers rather than proven causal determinants.

The treatment strategy also deserves emphasis. Corticosteroids are widely regarded as the first-line treatment for symptomatic organizing pneumonia and are usually tapered gradually, with most patients showing rapid clinical improvement [13]. However, this principle should be distinguished from the use of corticosteroids in acute severe influenza itself, where routine steroid administration has generally not shown benefit and may even be associated with harm. Therefore, the key clinical step is not merely administering steroids but identifying when the disease process has shifted from acute viral pneumonia to secondary organizing pneumonia [14]. In our patient, steroids were started together with antibiotics because bacterial pneumonia could not be fully excluded at presentation, and this was a pragmatic strategy given his age, imaging abnormalities, and ongoing deterioration. The subsequent response supports that an organizing component was clinically important. More fulminant pathological variants, particularly acute fibrinous and organizing pneumonia, may require more intensive management and carry a worse prognosis than typical organizing pneumonia, so recognizing the severity of the phenotype is also relevant when deciding on steroid dose and monitoring intensity [15].

Overall, this case reinforces that persistent or recurrent respiratory symptoms after influenza in older adults should prompt consideration of organizing pneumonia, especially when imaging shows bilateral subpleural or peribronchovascular infiltrates and the clinical course is not typical for bacterial lobar pneumonia. Early recognition of this entity may allow timely corticosteroid treatment, symptom resolution, and preservation of baseline function in vulnerable patients.

## Conclusions

This case suggests that postinfluenza organizing pneumonia should be considered in older adults with persistent or worsening respiratory symptoms after apparent recovery from influenza. In this patient, corticosteroid therapy was associated with clinical and radiological improvement; however, causality cannot be confirmed from a single case, particularly because antibiotics were administered concurrently and histological confirmation was not obtained. Further studies are needed to clarify the optimal diagnostic approach and treatment strategy for postinfluenza organizing pneumonia in older adults.
